# Exploring the Efficacy of Benzimidazolone Derivative as Corrosion Inhibitors for Copper in a 3.5 wt.% NaCl Solution: A Comprehensive Experimental and Theoretical Investigation

**DOI:** 10.3390/molecules28196948

**Published:** 2023-10-06

**Authors:** Mohamed Adardour, Mohammed Lasri, Marouane Ait Lahcen, Mohamed Maatallah, Rachid Idouhli, Mohamed M. Alanazi, Sanae Lahmidi, Abdesselam Abouelfida, Joel T. Mague, Abdesselam Baouid

**Affiliations:** 1 Laboratory of Chemistry Molecular, Department of Chemistry, Faculty of Sciences Semlalia, Cadi Ayyad University, B.P. 2390, Marrakech 40001, Morocco; m.aitlahcen.ced@uca.ac.ma (M.A.L.); m.maatallah@uca.ma (M.M.); baouid@uca.ac.ma (A.B.); 2 Applied Chemistry and Biomass Laboratory, Department of Chemistry, Faculty of Sciences Semlalia, Cadi Ayyad University, B.P. 2390, Marrakech 40001, Morocco; m.lasri.ced@uca.ac.ma (M.L.); rachid.idouhli@uca.ac.ma (R.I.); abouelfida@uca.ac.ma (A.A.); 3 Department of Pharmaceutical Chemistry, College of Pharmacy, King Saud University, P.O. Box 2457, Riyadh 11451, Saudi Arabia; mmalanazi@ksu.edu.sa; 4 Laboratory of Heterocyclic Organic Chemistry, Department of Chemistry, Faculty of Sciences, Mohammed V University in Rabat, Rabat 10106, Morocco; lahmidi_sanae@yahoo.fr; 5 Department of Chemistry, Tulane University, New Orleans, LA 70118, USA; joelt@tulane.edu

**Keywords:** benzimidazolone, corrosion inhibitor, electrochemical techniques, adsorption

## Abstract

This study focuses on the synthesis, theoretical analysis, and application of the corrosion inhibitor known as benzimidazolone, specifically 1-(cyclohex-1-enyl)-1,3-dihydro-2*H*-benzimiazol-2-one (CHBI). The structure of CHBI was determined by X-ray diffraction (XRD). The inhibitory properties of CHBI were investigated in a 3.5 wt.% NaCl solution on pure copper using various electrochemical techniques such as potentiodynamic polarization curves (PDPs) and electrochemical impedance spectroscopy (EIS), as well as scanning electron microscopy with energy dispersive X-ray spectroscopy (SEM-EDX), UV-visible spectroscopy, and theoretical calculations. The obtained results indicate that CHBI is an excellent inhibitor, exhibiting remarkable effectiveness with an inhibition rate of 86.49% at 10^−3^ M. To further confirm the extent of adsorption of the inhibitory molecule on the copper surface, density functional theory (DFT) and Monte Carlo (MC) simulation studies were conducted. The results of this study demonstrate the synthesis and characterization of CHBI as a corrosion inhibitor. The experimental and theoretical analyses provide valuable insights into the inhibitory performance of CHBI, indicating its strong adsorption on the copper surface.

## 1. Introduction

Organic inhibitors are an exceptionally effective and cost-efficient approach in the wide range of methods employed for corrosion prevention. This approach has gained widespread application for mitigating copper corrosion in multiple fields. Reviewing the existing literature unveils that the existence of heterocyclic compounds acts as active adsorption sites for facilitating the interaction between the corrosion inhibitor and copper through the exchange of electrons. In addition, heterocyclic compounds within aromatic structures exhibit effective corrosion inhibitory properties in a variety of corrosive environments [1,2,3,4].

Benzimidazolone is a heterocyclic aromatic organic compound consisting of the fusion of benzene and imidazolone rings. This heterocyclic framework serves as the foundational structure for a significant class of compounds, showcasing intriguing biological activities across a wide range of therapeutic domains. Recent research has found that benzimidazolone derivatives exhibit anti-HBV activities [5,6] as well as anticonvulsant [7] and antimicrobial [8] activities. Additionally, benzimidazole derivatives also play an important role in cancer treatment [9,10]. Benzimidazole derivatives featuring heteroatoms such as sulfur, nitrogen, and oxygen have emerged as some of the most widely employed corrosion inhibitors for various aggressive environments, particularly as effective coatings [11,12,13]. Benzimidazolone derivatives have a wide range of potential applications, including pharmaceuticals, inhibitors, herbicides, pigments, and fine chemicals [14]. In addition, benzimidazoles, are also of interest in advanced materials science, such as nonlinear optics [15]. Furthermore, due to the structure of benzimidazole, it can form hydrogen bonds with enzymes and biological receptors, take participate in π–π and hydrophobic interactions, and serve as a ligand for various metal ions.

**Figure 1:**
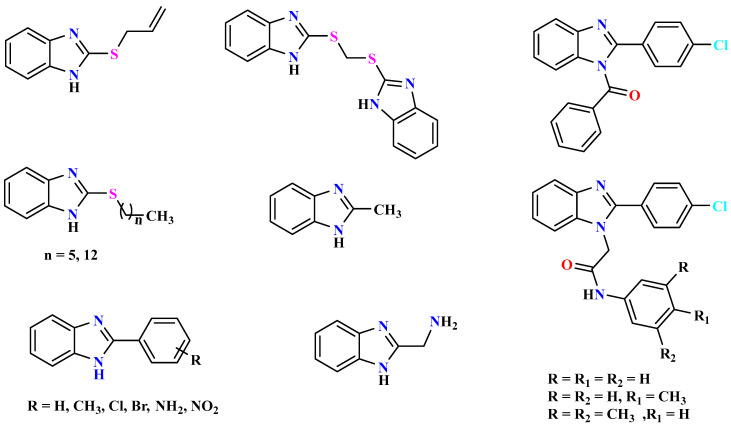
Structures of benzimidazole derivatives as corrosion inhibitors.

The literature abounds with examples that underscore the versatility and effectiveness of benzimidazole and its derivatives as corrosion inhibitors. Their unique molecular structure and compatibility with various industries and environments have solidified their role as essential components in ongoing efforts to protect valuable assets and infrastructure from the ravages of corrosion [16,17,18,19]. The use of benzimidazole compounds as corrosion inhibitors for copper at different concentrations of sodium chloride has been reported [20,21]. Recent experiments on corrosion [22,23,24,25] have shown that benzimidazole derivatives possess corrosion-inhibiting properties (Figure 1). According to the literature, most organic compounds are effective corrosion inhibitors, which are widely used to protect copper alloys against corrosion in aqueous environments [22,23,24,25,26,27,28]. Analogous compounds were employed as corrosion inhibitors for copper in a 3.5 wt.% NaCl solution at a temperature of 25 °C. The results suggest that introducing a nitrogen atom into the imidazole ring enhances the inhibitory effect, which is mainly due to the heightened adsorption energy. The three investigated inhibitors demonstrated a propensity to adsorb onto the copper surface in a parallel fashion, leading to optimal inhibition effects. This study holds the potential to introduce novel inhibitors with superior inhibitory performance and enhanced effects. Appa Rao et al. [29] documented the corrosion protection achieved by self-assembled monolayers of benzimidazole derivatives on copper surfaces immersed in a NaCl solution. Pareek et al. [30] investigated benzimidazole analogs as a copper corrosion inhibitor in 3.5 wt.% NaCl solution, using both experimental and theoretical methods. This compound has a corrosion inhibition efficiency of 92.79% at 0.80 mM.

Benzimidazole and its derivatives have also been employed as corrosion inhibitors for mild steel in an environment containing 1 M HCl [31,32]. These heterocyclic compounds are effective corrosion inhibitors for metals in various corrosive environments. In this context, the study carried out by Chaouiki [33] demonstrated the inhibition potential of certain benzimidazole compounds for mild steel in a HCl solution. The results showed that both benzimidazole derivatives acted as good corrosion inhibitors. Recently, Ech-chihbi et al. [34] conducted a study in which they examined organic compounds incorporating the benzimidazole ring. These heterocyclic compounds were assessed for their corrosion inhibition potential on mild steel in a 1 M HCl solution, utilizing both experimental and computational techniques. Within this context, the findings revealed that these compounds exhibited a substantial inhibition efficiency ($\eta_{EIS}$%) ranging from 90.4% to 95.7% at $10^{-4}$ M.

In the present study, the adsorption and inhibitive effects of CHBI on the corrosion of copper in a 3.5 wt.% NaCl solution were investigated. The corrosion inhibition effectiveness and adsorption characteristics of CHBI were examined through a combination of experimental methods, including Potentiodynamic Polarization analysis (PDP), Electrochemical Impedance Spectroscopy (EIS), and a morphological study using SEM-EDX. Additionally, a theoretical approach was employed, utilizing Density Functional Theory (DFT) calculations at the B3LYP/6–311G(d, p) level of theory, along with molecular dynamics simulation methods. These comprehensive methods provided a deeper understanding of CHBI’s corrosion inhibiting capabilities and its interactions with the copper surface.

## 2. Result and Discussion

### 2.1. Chemicals and Synthesis Procedure

**Scheme 1:**
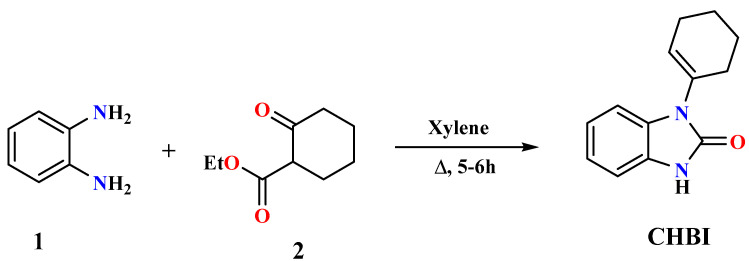
The synthesis procedure of CHBI.

The synthesis procedure for CHBI is illustrated in Scheme 1. It includes the condensation of o-phenylenediamine (**1**) and keto-ester (**2**) in refluxing xylene. The structure of CHBI was determined by analytical and spectroscopic analyses. The known CHBI was identified based on the spectroscopic data reported in the literature [35,36]. The structure of CHBI was confirmed by XRD measurements on single crystals as previously reported in another study [37].

### 2.2. X-ray Structure Description and Optimized Geometry

Specific information regarding the X-ray crystal data, structure, and refinement of CHBI is presented in Table 1. Supplementary data related to the compound are available and have been deposited with the Cambridge Crystallographic Data Center (CCDC) under the accession number **2280269**. Suitable crystals were grown by a slow evaporation of an ethanolic and dichloromethane (2:1, *v*/*v*) solution of the compound. The benzimidazolone moiety in CHBI is planar to within 0.0136(7) Å with N2 above the distance from the mean plane. A crystallographic analysis revealed that the cyclohexenyl ring in the compound is disordered and adopts two opposite conformations in a ratio of 0.5154(17)/0.4846(17). To account for this disorder, both components were refined with restraints applied to ensure that their geometries remained comparable during the refinement process. These restraints help to accurately represent the disorder in the cyclohexenyl ring and provide a comprehensive description of the molecule’s structure.

**Figure 2:**
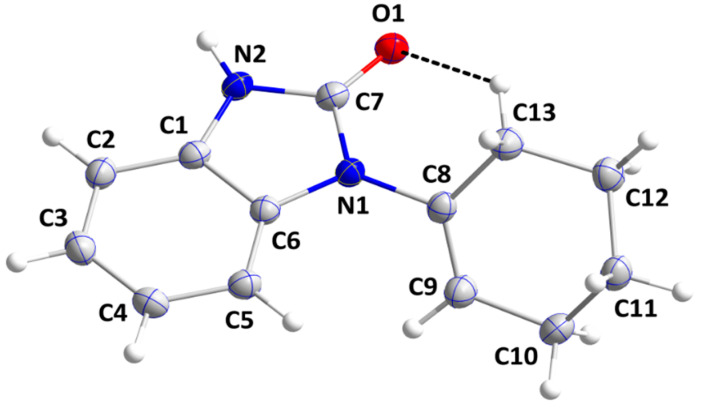
ORTEP view of the molecular structure of CHBI. The intramolecular hydrogen bond is depicted by a dashed line. Only the major component of the disordered cyclohexenyl ring is shown.

**Figure 3:**
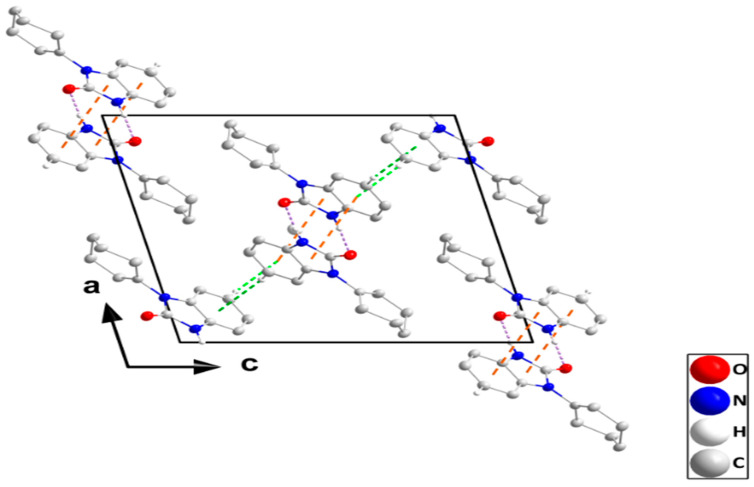
Detail of the intermolecular interactions in a portion of one chain. N—H···O and C—H···O hydrogen bonds are depicted, respectively, by violet and black dashed lines while C—H···π(ring) and π-stacking interactions are shown, respectively, by green and orange dashed lines.

A perspective view of CHBI is shown in Figure 2. In the crystal, paired N2–H2A···O1 hydrogen bonds (Table 2) form inversion dimers that are connected by C4–H4···Cg2 interactions (Table 2) and slipped π-stacking interactions between C1···C6 and C1/C6/N1/C7/N2 rings across inversion centers resulting in a layered structure, as shown in Figure 3.

### 2.3. Electrochemical Measurement

#### 2.3.1. Potentiodynamic Polarization

**Figure 4:**
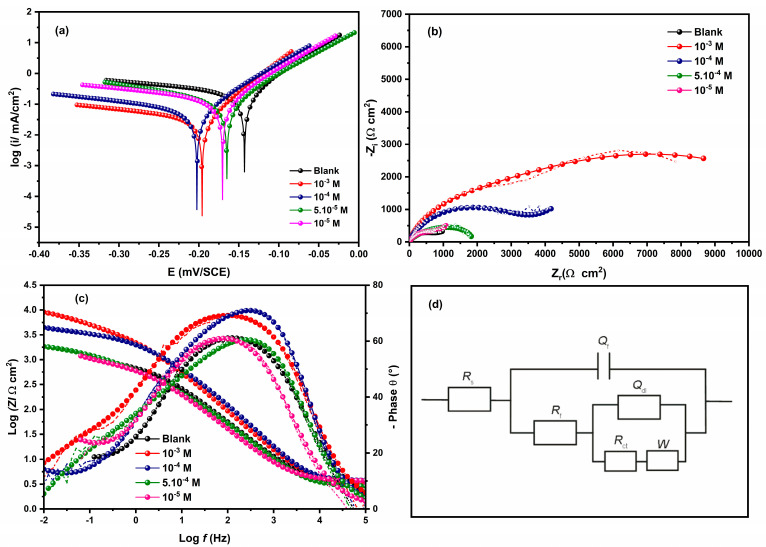
Polarization potentiodynamic curves (**a**), the electrochemical impedance Nyquist (**b**), and bode plots (**c**) of copper in 3.5 wt.% NaCl solution with different doses of CHBI for a 1 h immersion at 298 K. The circuit equivalent used (**d**).

Polarization curves for copper in a corrosive aqueous NaCl solution (3.5 wt.%) without and with different doses of CHBI are shown in Figure 4a, and the electrochemical parameters adjusted by Tafel analysis are given in Table 3, which include $E_{corr}$ (corrosion potential), $i_{corr}$ (corrosion current density), βa (anodic Tafel slope), βc (cathodic Tafel slope), and $n_{1}$% (corrosion inhibition efficiency). The corrosion inhibition efficiency was determined from the polarization results and can be used to calculate the corrosion inhibition capacity according to Equation (1), in which $i_{corr}^{{}^{\circ}}$ and $i_{corr}$ represent the current densities without and with the inhibitor, respectively.

(1) $$n_{1}\;\%=(1-\frac{i_{corr}}{{i{}^{\circ}}_{corr}})\times 100$$

In the absence of an inhibitor, a higher current density value is observed, which is explained by the anodic oxidation of Cu to ${Cu}^{+}$, which forms a soluble complex ${CuCl}_{2}^{-}$ with ${Cl}^{-}$ ions as shown in the following equations (Equations (2)–(4)) [38,39,40,41]:

(2) $$Cu\to {Cu}^{+}+e^{-}$$

(3) $${Cu}^{+}+{Cl}^{-}\to CuCl\;insoluble\;complex$$

(4) $$CuCl+{Cl}^{-}\to {CuCl}_{2}^{-}\;soluble\;complex$$

On the cathodic side, we observe the irreversible oxygen reduction controlled by charge transfer as shown below (Equation (5)) [42,43].

(5) $$O_{2}+2H_{2}O+{4e}^{-}\to {4OH}^{-}$$

As depicted in Figure 4a, the corrosion potential ($E_{corr}$) of the copper electrode changed negatively when the inhibitor CHBI was added, compared to that of the blank solution. Furthermore, an interesting observation is that the cathodic branches of the polarization curves remained parallel to the blank solution, suggesting minimal alterations in the cathodic reaction. However, the anodic branches displayed more pronounced variations when the inhibitor was introduced at different concentrations. This observation suggests that CHBI selectively affects the anodic corrosion reactions, highlighting its potential as a corrosion inhibitor for copper under the examined conditions. Based on the data presented in Table 3, with rising inhibitor concentration, the corrosion inhibition efficiency ($\eta_{1}\%$) shows an upward trend. The largest inhibition efficiency observed was 86.47% at 1 mM/L, indicating that a higher inhibitor concentration results in greater corrosion protection for copper surfaces. This suggests that the inhibitor can rapidly attach to the active sites on the copper surface during the 1-h immersion period, resulting in the formation of an anchored barrier film. This film effectively delays the redox reaction and exerts an active protective effect on the copper surface, contributing to the enhanced corrosion inhibition observed at higher inhibitor concentrations [44,45].

#### 2.3.2. Electrochemical Impedance Spectroscopy

The EIS was utilized to analyze the adsorption, protective effect, and kinetics of the corrosion process facilitated by CHBI on the copper surface. To investigate the effect of the inhibitor concentration on the inhibition efficiency, the inhibitor concentration was varied from 10^−3^ to 10^−5^ mol/L. The efficiency of the corrosion protection efficiency was determined from the results of the measurements of EIS ($n_{2}$%), which can be used to calculate the corrosion protection ability according to Equation (6), in which $R_{p}^{{}^{\circ}}$ and $R_{p}$ indicate the polarization resistances without and with inhibitor, respectively.

(6) $$n_{2}\;\%=(1-\frac{{R{}^{\circ}}_{p}}{R_{p}})\times 100$$

In Figure 4b, the Nyquist diagrams illustrate the impedance behavior of CHBI. The charge transfer resistance ($R_{ct}$) observed in the high-frequency loop of the Nyquist diagrams characterizes the mass transfer from the electrode surface, which occurs due to diffusion [46,47]. Meanwhile, the Warburg impedance (W) observed in the low-frequency region signifies the mass transfer from the electrode surface, which is also attributed to diffusion processes [48,49]. These EIS measurements provide valuable insights into the interactions between CHBI and the copper surface, shedding light on the inhibition efficiency of different inhibitor concentrations. The EIS data were analyzed using an equivalent circuit model, as illustrated in Figure 4d, and the corresponding parameters were calculated, as presented in Table 4. The circuit model comprises two Constant Phase Elements (CPE) connected in series with the uncompensated resistance $R_{s}$. This combination represents both the solution resistance and the resistance attributed to the electrical connections within the system. By utilizing this circuit model, the EIS analysis provides valuable information about the behavior of CHBI on the copper surface, offering insights into the inhibitive action and kinetics of the corrosion process at different inhibitor concentrations [50]. $R_{ct}$ represents the charge transfer resistance of copper dissolution between the metal surface and the external Helmholtz plane at the metal–solution interface. The Nyquist plots show an apparent deviation from the ideal semicircle, which is attributable to surface inhomogeneity due to surface roughness, impurities, and inhibitor adsorption [48].

In EIS, a CPE is frequently employed in place of an ideal capacitor to accommodate the frequency dispersion response [51]. In this context, the ${CPE}_{dl}$ is utilized to model the double-layer capacitance at the metal–solution interface. Additionally, the ${CPE}_{f}$ denotes the CPE associated with the surface film, where $R_{f}$ represents the corresponding film resistance. Moreover, the Warburg element (W) is employed to describe the diffusion mass transfer from the metal surface. By incorporating these elements in the equivalent circuit model, EIS provides a more accurate representation of the frequency-dependent behavior and facilitates a comprehensive analysis of corrosion inhibition and surface film formation on the copper electrode.

In Figure 4c, the phase angle versus log f (frequency) curves demonstrate a gradual increase in phase angle values, which approach approximately −75°. This behavior is typical of a non-ideal capacitor [52]. In Figure 4a,b, the curves of log |Z| (modulus of impedance) versus log f curves exhibit three distinct sections: (i) the high-frequency region, where the log |Z| values are smaller and remain nearly constant while the phase angle approaches zero. (ii) The medium-frequency region, which exhibits a linear relationship between log |Z| and log f values, and the phase angle reaches its maximum value. (iii) The low-frequency region, in which the log |Z| values become independent of log f, indicating the presence of Warburg impedance. This points toward a diffusion-controlled reactant transfer from the bulk electrolyte to the metal/electrolyte interface or transfer of soluble species from the interface to the bulk solution. Notably, the significantly higher phase-angle values observed in the presence of CHBI, compared to its absence, reflect its inhibitory performance. This suggests that CHBI effectively impedes the corrosion process, resulting in a noticeable alteration in the impedance behavior. This alteration can be attributed to the formation of a protective barrier on the surface of the copper.

#### 2.3.3. Adsorption Isotherm

**Figure 5:**
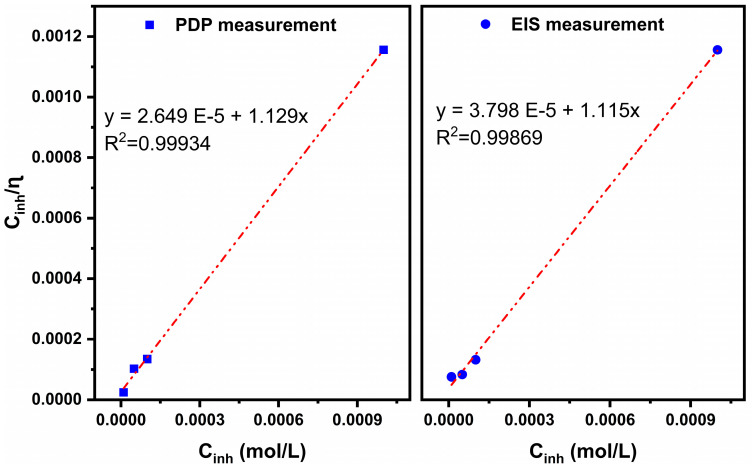
Langmuir adsorption curves of copper in 3.5 wt.% NaCl solution with different doses of CHBI.

The ability of organic corrosion inhibitors to adhere to metal surfaces is one factor that affects how much corrosion is reduced. Adsorption isotherms provide much essential information [53]. Like the adsorption isotherms (Langmuir), in this section we used the Langmuir adsorption isotherm using the corrosion inhibition efficiency values provided by the polarization potentiodynamic and EIS [54]. The Langmuir adsorption isotherm is a straight-line plot of C/θ with respect to the corrosion inhibitor concentration, as shown in Figure 5. The adsorption constant values ($K_{ads}$) of the inhibitor used were estimated according to the following equation (Equation (7)) and are given in Table 5.

(7) $$\frac{C_{inh}}{ɳ}=\frac{1}{K_{ads}}+C_{inh}$$

where ɳ is the surface coverage, *C* is the inhibitor concentration, and $K_{ads}$ is the adsorption equilibrium constant.

The Langmuir isotherm plots of CHBI are shown in Figure 5. The plots of C/ɳ as a function of *C* for pure copper in a 3.5wt.% solution at 298 K show a straight line. The linear correlation coefficient with *R*^2^ > 0.999 confirms that the Langmuir isotherm adequately accommodates the experimental data. The values of $K_{ads}$ are used to calculate the values of the change in adsorption standard free energy (${\Delta G}_{ads}^{{}^{\circ}}$) using Equation (8) [55]:

(8) $${\Delta G}_{ads}^{{}^{\circ}}=-RTLn(K_{ads})$$

where *R* is the universal gas constant and *T* is the absolute temperature (K).

Table 5 shows the values of $K_{ads}$ and ${\Delta G}_{ads}$ calculated from the EIS and PDP measurements. The adsorption process is more spontaneous when ${\Delta G}_{ads}$ is negative [56]. ${\Delta G}_{ads}$ values of 20 kJ/mol or less are typically associated with physisorption, while values of 40 kJ/mol or more are associated with chemisorption [57]. The values of ${\Delta G}_{ads}$ in this study indicate both chemisorption and physisorption, with a predominance of chemisorption.

#### 2.3.4. Temperature Influence

**Figure 6:**
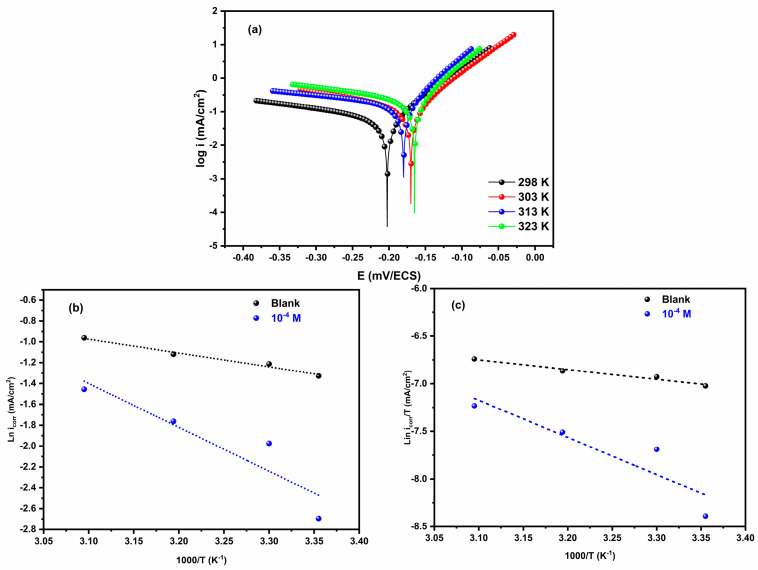
Polarization potentiodynamic curves (**a**) at different temperatures, Plots of Arrhenius (**b**) and Transition state (**c**) of copper in 3.5 wt.% NaCl without and with 7.5× 10^−4^ M of CHBI.

Electrochemical investigations were conducted to explore the effect of temperature on the corrosive degradation of pure copper when immersed in a 3.5 wt.% NaCl solution containing an inhibitor. The temperature range studied was between 298 and 323 K, employing PDP methods, as shown in Figure 6a. As shown by the outcomes in Table 6, both the corrosion current density values, with and without the inhibitor, exhibit an increase as the temperature rises. This rise in temperature leads to a shift in the inhibitor adsorption equilibrium, favoring a desorption process and consequently leading to a reduction in surface coverage [58,59]. As a result, the inhibitor surface conformation order decreases and the corrosive ion mobility increases [60]. At 323 K, the CHBI has a significant corrosion inhibition efficiency (38.88%) when compared to a blank, as shown in Table 6.

The inhibitor protection mechanism is based on thermodynamic activation characteristics [61]. The Arrhenius Equation (9) was used to calculate the corrosion activation energy ($E_{a}$) in 3.5 wt.% NaCl with and without CHBI:

(9) $$i_{corr}=Ae^{-\frac{Ea}{RT}}$$

where *T*, *A*, and *R* denote the absolute temperature, the Arrhenius factor, and the universal gas constant, respectively.

The CHBI activation energy ($E_{a}$) increased from 11.00 kJ.mol^−1^ to 34.95 kJ.mol^−1^ as measured by the Arrhenius diagrams in Figure 6b. The energy barrier for the corrosion reaction was increased, which improved corrosion inhibition [62]. A decrease in inhibition efficiency with rising temperature and a rise in $E_{a}$ when the inhibitor is present serve as evidence that a physical (electrostatic) adsorption film was formed (Table 7).

#### 2.3.5. Morphological Study by SEM-EDS

A polished circular section of copper, measuring 0.5 cm^2^ in area, was submerged for a period of 24 h at a temperature of 298 K in a 3.5 wt.% NaCl solution. This process was carried out both without and with the addition of 1mM of inhibitor (CHBI). After immersion, the surface homogeneity of the copper was meticulously inspected under both conditions to assess the extent of the reduction in NaCl activity in the presence of the inhibitor. The samples were analyzed by SEM for surface examination after being washed with doubly distilled water and dried. Furthermore, the atomic percentage of the elements was investigated using an EDX analyzer due to the inhibitor deposited on the copper surface [30].

To comprehend the inhibitory effect, it is crucial that the organic molecule forms a strong bond with the metal substrate, resulting in the formation of a protective layer on the metal surface [63]. SEM and EDX experiments were performed to see if the CHBI molecule actually gets adsorbed onto the copper surface. By examining and determining if CHBI is present on the copper surface, these analytical approaches can shed important light on how the inhibitor interacts with the metal.

**Figure 7:**
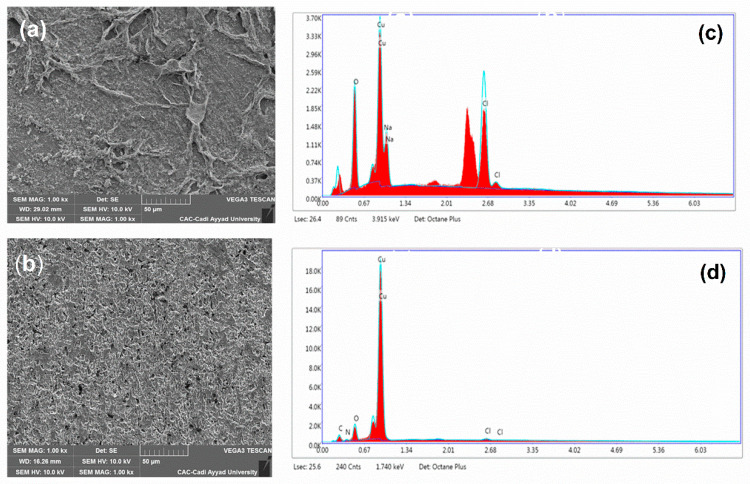
SEM images of copper in 3.5 wt.% NaCl (**a**) and in the presence of CHBI (**b**), Spectrum EDX of surface copper in NaCl solution without (**c**) and with CHBI (**d**) after 24 h of immersion at 298 K.

Figure 7a illustrates the copper surface after immersion in a 3.5 wt.% NaCl solution, revealing flaws such as pits, pores, and fractures caused by the dissolution of copper. The SEM micrograph of the surface immersed in NaCl for 24 h with 10^−3^ M of CHBI, which is shown in Figure 7b, exhibits the formation of a thin protective layer due to the adsorption of CHBI on the copper surface. According to our results, the presence of CHBI reduced the damage caused by cracking and pitting on the copper surface.

In the EDX spectrum (Figure 7c) of unprotected copper in the presence of the corrosive medium, we notice a peak for copper and another for oxygen, as well as other peaks that can be attributed to the generation of chlorides and sodium. Figure 7d shows the EDX spectrum of the surface in the presence of CHBI, and we observe peaks corresponding to carbon, nitrogen, and oxygen, which is explained by the formation of a protective film on the surface of the copper.

### 2.4. Results of Density Functional Theory and Molecular Dynamics Simulations

#### 2.4.1. Analysis of Conceptual DFT Indices

**Figure 8:**
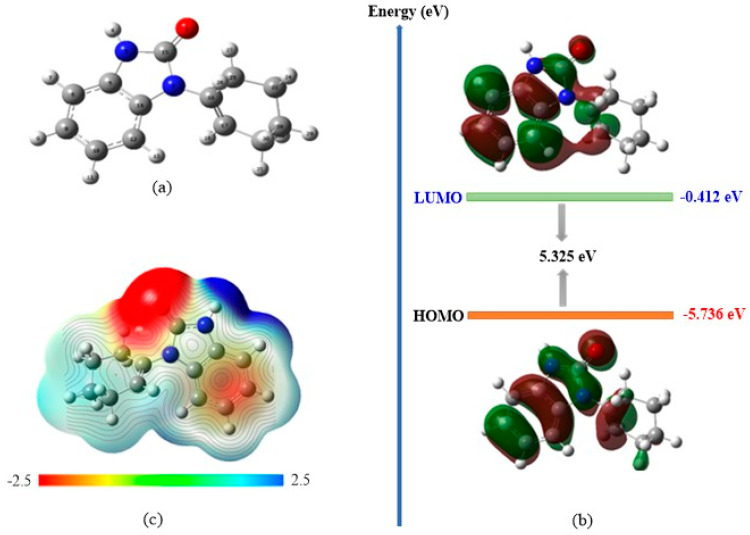
Optimized geometry (**a**), molecular electrostatic potential (MEP) (**c**), Plots of the frontier molecular orbitals and the HOMO-LUMO gap energy computed at the DFT level (**b**) of the theory for CHBI.

The structure of CHBI in its most stable configuration was determined by optimization at the 6-311G(d,p) level (Figure 8a). The calculated free enthalpy (−432,062.71577 Kcal/mol) indicates the thermodynamic stability of the molecule. After optimization, different quantum chemical descriptors characterizing the reactivity of CHBI were calculated using the above equations (Table 8).

The data in Table 8 indicate that CHBI has a high reactivity and a consistently good performance as an inhibitor. Considering the difference in electronegativity, CHBI easily interacts with the metal surface and can resist change or deformation in it since it exhibits high global hardness (2.662 eV) and low global softness (0.376 eV) values.

The interaction between CHBI and the surface is facilitated by a donation of electrons, which takes place from CHBI to the metal (∆N = 0.74 > 0). This result is supported by the energies of the HOMO (−5.736 eV) and the LUMO (−0.412 eV), which when compared with those of Cu [63] indicate a substantial interaction between the HOMO of CHBI and the LUMO of the metal, indicating that there is an electron transfer from CHBI to the empty orbitals of the metal.

The electron density distribution of the HOMO and the LUMO of CHBI, shown in Figure 8b, is located essentially on the CHBI ring including the N and O heteroatoms. Therefore, these atoms represent the most reactive centers of the CHBI and participate in its interaction with the metallic surface via their lone pairs. The interaction is further strengthened by the π electrons of the phenyl ring, and the values of w^−^ (3.64 eV), w^+^ (−1.96 eV), and ∆w ± (1.7 eV) also support the ability of CHBI to donate electrons to the metal surface.

#### 2.4.2. MEP Surface

The MEP is a very helpful tool for understanding how an inhibitor can interact with a metal surface by revealing its nucleophilic and electrophilic centers. The MEP is characterized by a colored zone according to the electrostatic potential. The maximum negative region (red areas) indicates electron-rich (nucleophilic) sites, while the maximum positive region (blue areas) characterizes electron-deficient (electrophilic) sites. Figure 8c shows the MEP of CHBI in its optimized form, through which the carbonyl oxygen (O1) and the phenyl ring (rich in π electron) are identified as electron-rich centers. These can interact with the metal surface as nucleophiles contributing electrons to its vacant sites. Similarly, the carbon and hydrogen atoms of the cyclohexenyl ring, where the blue zone is located, are likely to interact with the surface as electrophilic centers, accepting electrons from the electron-rich sites on the surface.

#### 2.4.3. Condensed Fukui Functions

The active sites and the local reactivity of the inhibitor molecule can also be understood using other descriptors such as the Fukui functions (f^+^, f^−^) where the electrophilic/nucleophilic character can be described as a function of the sign of the Fukui function. The values of the Fukui f^+^ and f^−^ functions are listed in Table 9. According to the table, the electrophilic attack site of CHBI was identified based on the positive charge value of the most reactive sites capable of accepting electrons during metal–inhibitor interactions. Furthermore, the CHBI Fukui function f^−^ illustrated the reactivity of the different areas within CHBI concerning the electrophilic reaction with electrophiles. A higher value of f^−^ suggested a more reactive site in those regions, indicating a greater tendency for electrophilic attack and implying that the atom in that region is more likely to donate electrons. This shows that the atoms of C6 and C12 exhibit the maximum values of f^+^ (0.122 and 0.133, respectively), indicating that these atoms are most likely to admit electrons from the copper surface to form feedback bonds, thus enhancing the interaction between the metal surface and the inhibitor. The atoms O1, N2, N3, and C8 have the largest values of f^−^ (0.134, 0.022, 0.029, and 0.069, respectively), making these atoms the most nucleophilic and effective adsorption sites. These results indicate the ability of the inhibitor CHBI to provide electrons throughout the molecule and thus explain the significant inhibitory effect of this type of organic inhibitor in aqueous solution.

### 2.5. In Silico Approaches for Environmental Toxicity

The toxicity characteristic of the CHBI examined was assessed using log (IGC_50_), which indicates the concentration required to inhibit 50% of CHBI growth in aquatic species. The results of the toxicity test are provided in Table 10. When examining Table 10, it becomes apparent that the logarithmic value (IGC_50_) obtained for CHBI was smaller than the study’s maximum concentration (10^−3^ M). This result suggests that the compounds under investigation showed evidence of having corrosion-inhibiting abilities that are friendly to the environment.

### 2.6. Molecular Dynamics Results

The inhibitory effect of the CHBI near the Cu surface was theoretically investigated by Monte Carlo (MC) and MD simulations. This simulation was carried out to analyze the behavior of CHBI in its neutral and protonated forms on the surface of Cu(110) in vacuum and in the presence of the corrosive medium $\left\langle H_{2}O|{Na}^{+}|{Cl}^{-}\right\rangle$.

**Figure 9:**
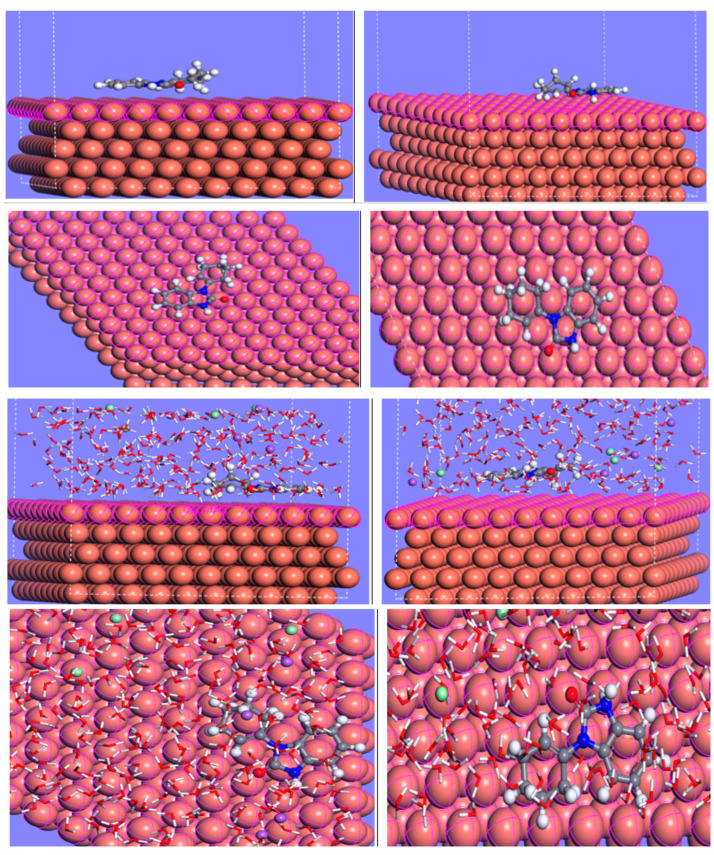
Side and top views of the most steady adsorption configurations for the adsorption of CHBI and protonated CHBI on Cu(110) surface (under vacuum and under H_2_O/NaCl).

The most stable equilibrium adsorption configurations of the neutral and protonated forms of CHBI on the Cu(110) surface are shown in Figure 9, and the various terms of the associated energy components are listed in Table 11. The data depicted in Figure 9 indicate that CHBI exhibits facile adsorption on the Cu(110) surface, adopting a parallel orientation. Such a position allows optimal interactions between heteroatoms and π-electrons with the metal surface, increasing surface coverage when interacting with the copper surface. This adsorption becomes stronger when the molecule is protonated (−77.08850781 and −98.97803808 Kcal/mol for the neutral and protonated forms, respectively), which is justified by the calculated energy values (Table 11). The parallel configuration of the molecule is also conserved in the presence of the corrosive medium. Based on the calculated energies listed in Table 12, the adsorption becomes stronger: −2255.320 and −2405.338 Kcal/mol for the two forms, respectively. These results are confirmed by the interaction and binding energies calculated under the equilibrium condition in an aqueous medium. The interaction and binding energies between the surface and the inhibitor are determined as follows (Equations (10) and (11)):

(10) $$E_{interaction}=E_{total}-E_{surface+solution}-E_{inhibitor}$$

(11) $$\begin{array}{cc} E_{interaction}= & {-E}_{Binding} \end{array}$$

where $E_{total}$ is the total energy of the entire system under study, $E_{surface+solution}$ represents the energy of the Cu(110) surface and solution, and $E_{inhibitor}$ is related to the energy of the inhibitor.

Table 13 presents the calculated $E_{interaction}$ and $E_{binding}$ values. The presence of negative $E_{interaction}$ values and positive $E_{binding}$ values indicates the spontaneous and robust adsorption process of both CHBI and protonated CHBI. An alteration in the trend of diminishing interaction and adsorption is noticed on the surface. Notably, higher values correspond to a better inhibition performance of the molecule. Therefore, from the calculated values, it can be concluded that our inhibitor readily adsorbs on the copper surface.

In the most stable equilibrium configurations, the aromatic ring of CHBI was aligned parallel to the Cu surface. Moreover, the oxygen and nitrogen atoms were oriented toward the metal surface as shown in Figure 9. This flat orientation is possibly due to the formation of coordination and back bonding between the Cu metal and CHBI inhibitor. It is herein evident that unoccupied copper orbitals (3d) will prefer to accept electrons from the adsorbed CHBI. In addition, CHBI has a lone pair of electrons on the active centers (NH, N, O), as well as π-electrons in the benzene ring. These electrons clouds provide sufficient electronic charges to the vacant orbitals (3d) of Cu and form stable coordination bonds (chemisorption). The spontaneous position of the CHBI inhibitor allows for maximum interactions between its active sites (the heteroatoms and the π electrons of the phenyl ring) and the metal surface. As a result, this increases its coverage and promotes the formation of a protective layer on the copper surface.

**Figure 10:**
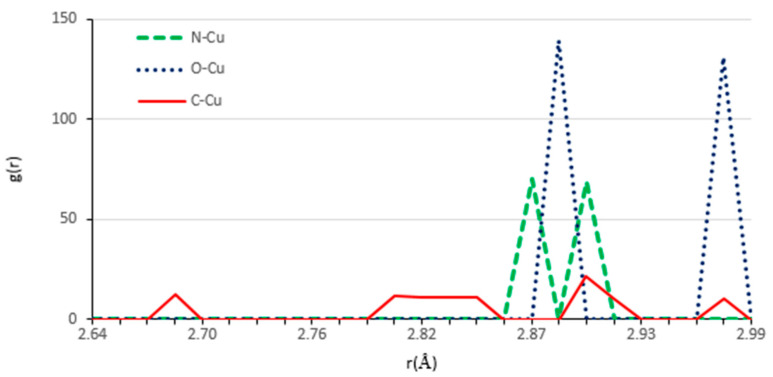
RDF analysis of CHBI adsorbed on the copper surface.

The formation of this monolayer suggests a chemisorption process between the inhibitor CHBI and the copper surface, which is consistent with the radial distribution function (RDF) results. The RDF was used to calculate the bond length between the atoms of CHBI and those on the Cu surface and to determine the type of bonds formed. Figure 10 shows the variations of the RDF as a function of bond length (r). The link length values varied from 2.68 to 2.98 Å, which indicates that the interactions developed are of the chemisorption type.

## 3. Materials and Methods

### 3.1. Chemistry

The synthesis of CHBI was successfully accomplished following the steps outlined in Scheme 1. All the chemical reagents utilized in the synthesis were obtained from Sigma Aldrich (St. Somaprol, Morocco) and were used without any further purification. The purity of CHBI inhibitor was determined by thin-layer chromatography (TLC) and melting point (m.p). IR spectra were recorded on a Bruker Vertex 70 spectrometer as potassium bromide discs. Melting points were taken in an open capillary tube on a Buchi 510 apparatus and were uncorrected. The spectra were recorded with the following instruments: ^1^H NMR (AC-300) and ^13^C NMR (AC-75) spectra were recorded on Bruker spectrometers (Bruker, France) with chemical shift values (d) given in part per million (ppm) relative to TMS (0.00 ppm). Mass spectra: Jeol JMS DX 300. TMS was used as an internal reference. Elemental analysis was performed on a EuroEA Elemental Analyzer.

#### Synthesis of CHBI

The CHBI was prepared according to a reported procedure [37]. A mixture of o-phenylenediamine **1** (23 mmol) and ketoester **2** (26 mmol) in absolute xylene. The reaction was carried out at a reflux temperature for a duration of 5 h (Scheme 1). The reaction mixture was then cooled, and the precipitated solid was filtered and recrystallized from ethanol to obtain CHBI. The CHBI was obtained in the form of a white solid. Yield: 92%. m.p: 181–183 °C (ethanol). IR (KBr, ν (cm^−1^), 3413 (imidazole-NH), 1602 (C=O). ^1^H NMR (300 MHz in CDCl_3_) (δ ppm): 1.69, 1.78, 2.22, 2.33 (m, 8H, 4CH_2_), 5.90 (m, 1H, =CH), 6.89–7.17 (m, 4H, H-Ar), 10.77 (s, 1H, NH). ^13^C NMR (75 MHz in CDCl_3_) (δ ppm): 21.65, 22.60, 24.76, 26.87 (4CH_2_), 127.76 (1C, =CH), 108.71, 110.09, 121.38, 121.69 (CH-Ar), 128.44, 130.54, 132.07 (=C), and 154.06 (C=O). HRMS of [M+H]+ *m*/*z* 215.1179, calcd for C_13_H_14_N_2_O, 215.1140. Elem. Anal. Calcd for C_13_H_14_N_2_O: C 72.87%, H 6.59%, N 13.07%. Found: C 71.63%, H 6.53%, N 13.54%.

### 3.2. X-ray Analysis

The collection of single-crystal X-ray data was performed using a Bruker D8 VENTURE PHOTON 3 CPAD diffractometer with Cu-Kα radiation (λ = 1.54178 Å) at a temperature of 150 K. The intensity data obtained were converted to F^2^ values using SAINT [64]. Subsequently, a multi-scan absorption correction was applied, and the equivalent reflections were merged using SADABS [65]. The structure was initially solved using dual-space methods with SHELXT [66]. Afterward, full-matrix, least-squares procedures were employed to refine the structure using SHELXL [67]. Hydrogen atoms bonded to carbon were incorporated as riding contributions in idealized positions, with their isotropic displacement parameters linked to those of the attached atoms. This approach is commonly used in crystallographic refinement to simplify the process and improve accuracy. The hydrogen atom attached to nitrogen was initially identified in a difference map and further refined using the DFIX 0.91 0.01 instruction. The specifics of the X-ray crystal data, the structural solution, and the refinement process are all presented in Table 1. This table likely contains the relevant details necessary for understanding the crystallographic analysis and the determined structure.

### 3.3. Corrosion Methods

#### 3.3.1. Preparation of Materials

In this experiment, a highly pure copper electrode (with 99.9% purity) was immersed in a 3.5 wt.% NaCl solution, which served as a highly corrosive environment. To protect the copper from corrosion, CHBI was used as a corrosion inhibitor at various concentrations: 1, 0.1, 0.05, and 0.01 mM. Before conducting the tests, CHBI was introduced into a blank solution. Prior to the corrosion testing, the copper samples underwent a step-by-step polishing process using different grades of emery paper (ranging from 400 to 2500 grains). The samples were then rinsed with distilled water and anhydrous ethanol and left to dry at room temperature. The assessment of CHBI’s corrosion inhibition effectiveness was conducted by immersing the copper samples in the corrosive solution for a duration of 1 h. This allowed the researchers to evaluate the inhibitory performance of CHBI under the specified conditions.

#### 3.3.2. Electrochemical Measurements

The electrochemical assessments were conducted using the potentiostat/galvanic PGZ 100 electrochemical workstation, employing the conventional three-electrode system [68]. For this setup, a pure copper electrode with an effective exposed area of 0.5 cm^2^ was utilized as the working electrode. Additionally, a saturated calomel electrode (SCE) was employed as the reference electrode, and a platinum electrode with a surface area of 1 cm^2^ was used as the counter electrode. A 3.5 wt.% NaCl solution was applied to the working electrode for 1 h at 298 K. Following the immersion, two electrochemical tests were conducted: EIS and PDP measurements. For the PDP measurement, the polarization curve was scanned from −1000 mV to +600 mV using a scan rate of 1 mV s^−1^. On the other hand, the EIS was performed across a frequency range spanning from 0.01 Hz to 100 kHz, with an amplitude of 10 mV [69,70]. The EC-Lab software (V10.44) package was utilized to analyze the electrochemical data, employing equivalent electrical circuits and associated fitting parameters. To ensure the reliability of the results, a minimum of three repeated measurements were carried out, aiming to attain good reproducibility.

### 3.4. Theoretical Study Computational Details

#### DFT Study

To understand the performance of CHBI from an electronic point of view, structural optimization was performed, and the electronic properties of CHBI were obtained using DFT implemented in Gaussian 09 [71] with the hybrid B3LYP functional and the 6–311G(d, p) basis set [72,73,74]. The optimization was followed by a frequency calculation to ensure that the optimized structure is at the minimum on the potential energy surface.

The frontier molecular orbitals (FOM), Highest Occupied Molecular Orbital (HOMO), and Lowest Unoccupied Molecular Orbital (LUMO) play a crucial role in the analysis of the chemical reactivity of organic molecules. They are also employed to investigate the inhibitory properties of molecules, as their adsorption is associated with the potential interaction between the HOMO and LUMO of the inhibitory molecule and the metal surface. The mode of interaction can be determined by a transfer of electrons, either by a donation of electrons from the molecule toward the metal, or by a retro-donation of electrons from the metal toward the inhibitor. For this purpose, based on the energies of the frontier orbitals, the reactivity of the inhibitor was studied from quantum chemical parameters such as the band gap (∆Eg), the ionization potential (I), the electron affinity (A), the hardness (η), the softness (σ), and the electronegativity (χ). The electron transfer fraction (∆N) and the electrophilicity index (ω) were also evaluated since these are two characteristics responsible for the possible electronic exchange between the molecules and the surface. The Fukui parameters serve as valuable descriptors that can be utilized to identify the reactive centers involved in the inhibition process. These descriptors cited above were calculated based on the following expressions (Equations (12)–(18)) [75,76,77]:

(12) $$∆E_{gap}=E_{LUMO}-E_{HOMO}$$

(13) $$µ=\frac{I-A}{2}\;\mathrm{and}\;\chi =-\frac{1}{2}\left(E_{HOMO}+E_{LUMO}\right)$$

(14) $$\eta =\frac{I-A}{2}\;\mathrm{and}\;\sigma =\frac{1}{\eta }$$

(15) $$I=-E_{HOMO}\;{\mathrm{and}}_{\;}A=-E_{LUMO}$$

(16) $$N=E_{HOMO}-E_{HOMO(TCE)}\;\mathrm{and}\;\omega ={\frac{\mu }{2\eta }}^{2}$$

(17) $$f^{+}=q_{k}\left(N+1\right)-q_{k}\left(N\right)\;For\;nucleophilic\;attack$$

(18) $$f^{-}=q_{k}\left(N\right)-q_{k}\left(N+1\right)\;For\;electrophilic\;attack$$

### 3.5. Toxicity Approach

In silico research was performed using a chemical database and modeling environment website called OCHEM to assess the toxicity of the CHBI molecule. Two subsystems make up this website: a main database of experimental techniques, and a modeling framework [78,79].

### 3.6. Molecular Dynamic Simulation Method

A molecular dynamics (MD) simulation was performed between the inhibitor molecule and the Cu(110) surface using a slab of 5 Å. Simulation boxes of dimensions 25.50 × 25.50 × 38.33 Å^3^ were created, and a vacuum plate of 30 thickness was introduced. The MD simulations were carried out at a constant temperature of 300 K using the Andersen thermostat, a time step of 1 fs, an NVT set, and a simulation time of 2000 ps to reach the equilibrium configuration. Energy minimization and MD calculations in the condensed phase were performed using the Condensed-phase Optimized Molecular Potential for Atomistic Simulation Studies (COMPASS) force field.

## 4. Conclusions

In this study, we synthesized the compound CHBI for copper corrosion inhibition. We found that CHBI is an excellent inhibitor of copper corrosion in a 3.5 wt.% NaCl solution. The inhibition efficiency increases with inhibitor concentration and reaches a maximum of 86.49% at $10^{-3}$ M. The adsorption of CHBI on the copper surface follows the Langmuir adsorption isotherm. Increasing the temperature from 298 to 323 K decreases the inhibition efficiency of CHBI to 38%. MEB-EDX analysis revealed that the adsorption of CHBI on the copper surface forms a protective layer. DFT numerical simulation results showed that CHBI has a strong ability to donate electrons to the copper surface, which confirms its excellent anticorrosive activity. A strong correlation was observed between quantum chemical parameters and the experimentally obtained inhibition efficiencies of CHBI.

These findings not only expand our understanding of CHBI’s role as a corrosion inhibitor but also highlight potential avenues for further research in corrosion protection and inhibitor optimization, holding promise for practical applications in protecting copper and related materials from corrosion.

## Figures and Tables

**Table 1:** Crystallographic data and structure refinement summary for CHBI.

Empirical formula	C_13_H_14_N_2_O
Crystal system, space group	Monoclinic, P21/n
Temperature (K)	150
a, b, c (Å)	12.6343(4), 6.4043(2), 13.5951(4)
α, β, γ (°)	90, 103.7730(10), 90
V (Å^3^)	1068.40(6)
*Z* Density (g/cm^3^)	4 1.332
Absorption coefficient	0.684 mm^−1^
Crystal size (mm)	0.218 × 0.244 × 0.316
F(000)	456
Independent reflections	2051 [R(int) = 0.0181]
Data/restraints/parameters	2051/7/162
Goodness-of-fit on F^2^	1.066
R indices (all data)	R1 = 0.0338, wR2 = 0.0842
Largest diff. peak and hole	0.210 and −0.155 ${eÅ}^{-3}$

**Table 2:** Hydrogen-bond geometry (Å, °).

D—H···A	D—H	H···A	D···A	Angle
N2—H2O···O1 ^i^	0.925(15)	1.872(15)	2.7946(11)	176.1(13)
C13–H13B···O1	0.99	2.43	3.097(8)	124

Symmetry codes: (i) −x + 1, −y + 2, −z + 1.

**Table 3:** Electrochemical analysis using the technique of polarization potentiodynamic.

Concentration (mol/L)	$E_{corr}$ (mV/SCE)	$i_{corr}$ (mA/cm^2^)	*β*a mV/dec	*β*c mV/dec	$n_{1}$%
Blank	−146 ± 4	0.265 ± 0.007	76 ±7	−435 ± 3	-
10^−5^	−170 ± 3	0.156 ± 0.005	69 ± 4	−403 ± 6	41.10
5 × 10^−5^	−164 ± 5	0.135 ± 0.008	72 ± 6	−261 ± 8	49.01
10^−4^	−202 ± 4	0.067 ± 0.004	67 ± 5	−358 ± 4	74.51
10^−3^	−195 ± 2	0.035 ± 0.003	51 ± 8	−370 ± 9	86.47

**Table 4:** Adjusted parameters of EIS spectra for copper in 3.5 wt.% NaCl solution in the absence and presence of various doses of CHBI at 298 K.

Conc. (mol/L)	$R_{s}$ (Ω cm^2^)	$R_{f}$ (Ω cm^2^)	$R_{dl}$ (Ω cm^2^)	a	$Q_{f}$ (μS em^−2^)	$Q_{dl}$ (μS cm^−2^)	$R_{p}$ (Ω cm^2^)	*W* (mS cm^−2^)	${ɳ}_{2}$%
**Blank**	3.03	402.90	516.83	0.75	137.8	521.2	926.73	455.9	-
**10^−5^**	3.48	217.17	851.71	0.52	128.5	131.0	1068.88	279.8	13.29
**5** **×** **10^−5^**	2.72	228.64	2092	0.38	76.0	71.93	2320.64	148.9	60.06
**10^−4^**	3.73	239.77	3584	0.90	21.48	97.38	3823.77	226.3	75.76
**10^−3^**	3.12	518.14	6339	0.96	14.56	28.82	6857.14	118.6	86.49

**Table 5:** $K_{ads}$ and ${\Delta G}_{ads}^{0}$ for adsorption on copper in 3.5 wt.% NaCl solution containing different concentrations of CHBI.

	PDP Measurement			EIS Measurement		
	*R* ^2^	$$K_{ads}$$	${\Delta G}_{ads}^{{}^{\circ}}$ (kJ/mol)	*R* ^2^	$K_{ads}$	${\Delta G}_{ads}^{{}^{\circ}}$ (kJ/mol)
**CHBI**	0.99934	37.750 × 10^3^	−36.044	0.99869	26.329 × 10^3^	−35.151

**Table 6:** Electrochemical parameters at different temperatures of copper immersed in 3.5 wt.% NaCl solution with and without 10^−4^ M of CHBI.

	Temperature (K)	$E_{corr}$ (mV/SCE)	$i_{corr}$ mA/cm^2^	*β*a mV/dec	*β*c mV/dec	${ɳ}_{1}$ (%)
**Blank**	**298**	−146 ± 4	0.265 ± 0.007	76 ± 7	−435 ± 3	-
	**303**	−165 ± 2	0.297 ± 0.006	80 ± 4	−535 ± 6	-
	**313**	−154 ± 5	0.326 ± 0.008	62 ± 5	−695 ± 9	-
	**323**	−189 ± 2	0.381 ± 0.009	76 ± 2	−845 ± 6	-
**10^−4^ M**	**298**	−202 ± 4	0.067 ± 0.004	67 ± 5	−358 ± 4	74.51
	**303**	−169 ± 3	0.138 ± 0.003	65 ± 6	−291 ± 3	53.31
	**313**	−180 ± 2	0.171 ± 0.006	56 ± 3	−464 ± 1	47.48
	**323**	−165 ± 4	0.233 ± 0.001	58 ± 1	−373 ± 5	38.88

**Table 7:** Activation data of the corrosion reaction of copper in 3.5 wt.% NaCl with and without CHBI.

Milieu	$E_{a}$ (KJ/mol)	${\Delta H}_{ads}^{{}^{\circ}}$ (KJ/mol)	${\Delta S}_{ads}^{{}^{\circ}}$ (J/mol)
**Blank**	11.00	8.43	−201.05
**CHBI**	34.95	32.39	−192.54

**Table 8:** Quantum molecular descriptors for CHBI (eV).

HOMO Energy	$E_{HOMO}$	−5.736
LUMO Energy	$E_{LUMO}$	−0.412
HOMO-LUMO Gap energy	${∆E}_{g}$	5.325
Ionization Potential	*I*	5.736
Electron Affinity	*A*	0.412
Chemical hardness	η	2.662
Softness	σ	0.376
Electronegativity	χ	3.074
Fraction of electron transfer	∆N	0.737
Electrophilicity index	ω	1.775
Electrodonating power	$\omega^{-}$	3.644
Electroaccepting power	ω^+^	−1.959
Net electrophilicity	∆ω	1.686
Back-donation	${∆E}_{back-d}$	−0.666

**Table 9:** Fukui indices of the atoms in CHBI.

Atoms	Fukui Indices	Fukui Indices
	f^+^	f^−^
O1	0.075	0.134
N2	−0.005	0.022
N3	0.014	0.029
H4	0.031	0.039
C5	0.025	0.052
C6	0.122	0.028
H7	0.079	0.058
C8	0.048	0.069
H9	0.068	0.068
C10	0.029	0.051
H11	0.067	0.065
C12	0.133	0.028
H13	0.070	0.054
C14	0.025	0.038
C15	0.033	0.045
C16	0.005	0.007
C17	0.027	0.029
H18	0.027	0.030
C19	−0.018	−0.015
H20	0.033	0.034
H21	0.025	0.033
C22	−0.014	−0.013
H23	0.021	0.019
H24	0.030	0.032
C25	−0.015	−0.014
H26	0.017	0.024
H27	0.019	0.018
C28	−0.012	−0.013
H29	0.031	0.033
H30	0.011	0.016

**Table 10:** In silico value of log (IGC_50_) predicted by an online chemical data website.

Compound	Log (IGC_50_) in Log(mmol/L)
CHBI	0.98

**Table 11:** Calculated MC simulation parameters for adsorption of CHBI on a Cu(110) surface in vacuum.

Structure	Vacuum	
	CHBI	Protonated CHBI
Total energy	−96.62661	−146.19694
Adsorption energy	−77.08851	−98.97804
Rigid adsorption energy	−69.30039	−67.35659
Deformation energy	−7.78811	−31.62145
**CHBI: ** ${dE}_{ad}/{dN}_{i}$	−77.08851	−98.97804

**Table 12:** Calculated MC simulation parameters for adsorption of CHBI on a Cu(110) surface in H_2_O/NaCl.

Structure	Medium (H_2_O + NaCl)	
	CHBI	Protonated CHBI
Total energy	−2170.694	−2372.227
Adsorption energy	−2255.320	−2405.338
Rigid adsorption energy	−2255.320	−2405.338
Deformation energy	−7.109143 × $10^{-10}$	−3.629109 × $10^{-10}$
**H_2_O: ** ${dE}_{ad}/{dN}_{i}$	10.83717513	6.85686319
**Na: ** ${dE}_{ad}/{dN}_{i}$	−110.930651	−119.469374
**Cl: ** ${dE}_{ad}/{dN}_{i}$	−111.212166	−88.177719
**CHBI: ** ${dE}_{ad}/{dN}_{i}$	−65.9181022	−61.2905344

**Table 13:** Calculated $E_{interaction}$ and $E_{binding}$ (kJ/mol) values obtained for the inhibitor/Cu (110) surface.

	Medium (H_2_O + NaCl)	
	CHBI	Protonated CHBI
**E_interaction_** (Kj/mol)	−2081.85161	−1951.98325
**E_binding_** (Kj/mol)	2081.85161	1951.98325

## Data Availability

Not applicable.

## Supplementary Materials

The following supporting information can be downloaded at: https://www.mdpi.com/article/10.3390/molecules28196948/s1, Figures S1–S3: ^1^H-NMR, ^13^C- NMR and HRMS spectrums of CHBI.

## References

[1] Sarkar T.K., Saraswat V., Mitra R.K., Obot I.B., Yadav M. (2021). Mitigation of Corrosion in Petroleum Oil Well/Tubing Steel Using Pyrimidines as Efficient Corrosion Inhibitor: Experimental and Theoretical Investigation. Mater. Today Commun..

[2] Sarkar T.K., Yadav M., Obot I.B. (2022). Mechanistic Evaluation of Adsorption and Corrosion Inhibition Capabilities of Novel Indoline Compounds for Oil Well/Tubing Steel in 15% HCl. Chem. Eng. J..

[3] Hussien H., Shahen S., Abdel-karim A., El-Shamy O., Ghayad I., Mostfa N., Ahmed N.E.-D. (2023). Experimental and Theoretical Evaluations: Green Synthesis of New Organic Compound Bis Ethanethioyl Oxalamide as Corrosion Inhibitor for Copper in 3.5% NaCl. Egypt. J. Chem..

[4] Verma C., Abdellattif M.H., Alfantazi A., Quraishi M.A. (2021). N-Heterocycle Compounds as Aqueous Phase Corrosion Inhibitors: A Robust, Effective and Economic Substitute. J. Mol. Liq..

[5] Akbay A., Ören İ., Temiz-Arpacı Ö., Akı-Sener E., Yalcçın I. (2011). Synthesis and HIV-1 Reverse Transcriptase Inhibitor Activity of Some 2,5,6-Substituted Benzoxazole, Benzimidazole, Benzothiazole and Oxazolo(4,5-b)Pyridine Derivatives. Arzneimittelforschung.

[6] Qiu J., Zou Y., Li S., Yang L., Qiu Z., Kong F., Gu X. (2022). Discovery of Benzimidazole Substituted 1, 2, 4-Oxadiazole Compounds as Novel Anti-HBV Agents with TLR8-Agonistic Activities. Eur. J. Med. Chem..

[7] Sahoo B.M., Banik B.K., Mazaharunnisa, Rao N.S., Raju B. (2019). Microwave Assisted Green Synthesis of Benzimidazole Derivatives and Evaluation of Their Anticonvulsant Activity. CMIC.

[8] Ersan R.H., Yuksel A., Ertan-Bolelli T., Dogen A., Burmaoglu S., Algul O. (2021). One-pot Synthesis of Novel Benzimidazoles with a Naphthalene Moiety as Antimicrobial Agents and Molecular Docking Studies. J Chin Chem Soc.

[9] Rashid M., Husain A., Mishra R. (2012). Synthesis of Benzimidazoles Bearing Oxadiazole Nucleus as Anticancer Agents. Eur. J. Med. Chem..

[10] Karadayi F.Z., Yaman M., Kisla M.M., Keskus A.G., Konu O., Ates-Alagoz Z. (2020). Design, Synthesis and Anticancer/Antiestrogenic Activities of Novel Indole-Benzimidazoles. Bioorganic Chem..

[11] Tecuapa-Flores D., Guadalupe Hernández J., Alejandro Reyes Domínguez I., Turcio-Ortega D., Cruz-Borbolla J., Thangarasu P. (2022). Understanding of Benzimidazole Based Ionic Liquid as an Efficient Corrosion Inhibitor for Carbon Steel: Experimental and Theoretical Studies. J. Mol. Liq..

[12] Toukal L., Foudia M., Haffar D., Aliouane N., Al-Noaimi M., Bellal Y., Elmsellem H., Abdel-Rahman I. (2022). Monte Carlo Simulation and Electrochemical Performance Corrosion Inhibition Whid Benzimidazole Derivative for XC48 Steel in 0.5 M H2SO4 and 1.0 M HCl Solutions. J. Indian Chem. Soc..

[13] Salcı A., Yüksel H., Solmaz R. (2022). Experimental Studies on the Corrosion Inhibition Performance of 2-(2-Aminophenyl)Benzimidazole for Mild Steel Protection in HCl Solution. J. Taiwan Inst. Chem. Eng..

[14] Nale D.B., Bhanage B.M. (2015). Copper-Catalyzed Efficient Synthesis of a 2-Benzimidazolone Scaffold from 2-Nitroaniline and Dimethyl Carbonate via a Hydrosilylation Reaction. Green Chem..

[15] Rodembusch F.S., Buckup T., Segala M., Tavares L., Correia R.R.B., Stefani V. (2004). First Hyperpolarizability in a New Benzimidazole Derivative. Chem. Phys..

[16] Xu X., Qu J., Huang H. (2023). Synthesis of a Dibenzimidazole Compound and Its Corrosion Inhibition Behavior on AZ91D Mg Alloy in 3.5 Wt.% NaCl Solution. J. Mol. Struct..

[17] Shi N., Li H., Li X., Luo H., Jin J., Wang J., Li S. (2023). ZIF-8 and Benzimidazole Co-Modified h-BN for Enhancing Anti-Corrosion Performance of Epoxy Coatings. Prog. Org. Coat..

[18] Wang P., Xiong L., He Z., Xu X., Hu J., Chen Q., Zhang R., Pu J., Guo L. (2022). Synergistic Effect of Imidazoline Derivative and Benzimidazole as Corrosion Inhibitors for Q235 Steel: An Electrochemical, XPS, FT-IR and MD Study. Arab J Sci Eng.

[19] Marinescu M. (2019). Recent Advances in the Use of Benzimidazoles as Corrosion Inhibitors. BMC Chem..

[20] Eldebss T.M.A., Farag A.M., Shamy A.Y.M. (2019). Synthesis of Some Benzimidazole-based Heterocycles and Their Application as Copper Corrosion Inhibitors. J. Heterocycl. Chem.

[21] Wang X., Li W., Tan B., Wang F., Du H., Liu R., Han X., Zhang S. (2023). Corrosion Inhibition Effect of Benzimidazole and Two Derivatives on Copper in Alkaline Environments: Experimental and Theoretical Analyses. J. Mol. Liq..

[22] Lgaz H., Masroor S., Chafiq M., Damej M., Brahmia A., Salghi R., Benmessaoud M., Ali I.H., Alghamdi M.M., Chaouiki A. (2020). Evaluation of 2-Mercaptobenzimidazole Derivatives as Corrosion Inhibitors for Mild Steel in Hydrochloric Acid. Metals.

[23] Ech-chihbi E., Nahlé A., Salim R., Benhiba F., Moussaif A., El-Hajjaji F., Oudda H., Guenbour A., Taleb M., Warad I. (2020). Computational, MD Simulation, SEM/EDX and Experimental Studies for Understanding Adsorption of Benzimidazole Derivatives as Corrosion Inhibitors in 1.0 M HCl Solution. J. Alloys Compd..

[24] Fergachi O., Benhiba F., Rbaa M., Ouakki M., Galai M., Touir R., Lakhrissi B., Oudda H., Touhami M.E. (2019). Corrosion Inhibition of Ordinary Steel in 5.0 M HCl Medium by Benzimidazole Derivatives: Electrochemical, UV–Visible Spectrometry, and DFT Calculations. J Bio Tribo Corros.

[25] Damej M., Benmessaoud M., Zehra S., Kaya S., Lgaz H., Molhi A., Labjar N., El Hajjaji S., Alrashdi A.A., Lee H.-S. (2021). Experimental and Theoretical Explorations of S-Alkylated Mercaptobenzimidazole Derivatives for Use as Corrosion Inhibitors for Carbon Steel in HCl. J. Mol. Liq..

[26] Sabet Bokati K., Dehghanian C. (2018). Adsorption Behavior of 1H-Benzotriazole Corrosion Inhibitor on Aluminum Alloy 1050, Mild Steel and Copper in Artificial Seawater. J. Environ. Chem. Eng..

[27] Singh A., Ansari K., Quraishi M., Lgaz H. (2018). Effect of Electron Donating Functional Groups on Corrosion Inhibition of J55 Steel in a Sweet Corrosive Environment: Experimental, Density Functional Theory, and Molecular Dynamic Simulation. Materials.

[28] Lgaz H., Saha S.K., Lee H., Kang N., Thari F.Z., Karrouchi K., Salghi R., Bougrin K., Ali I.H. (2021). Corrosion Inhibition Properties of Thiazolidinedione Derivatives for Copper in 3.5 Wt.% NaCl Medium. Metals.

[29] Appa Rao B.V., Iqbal M.Y., Sreedhar B. (2010). Electrochemical and Surface Analytical Studies of the Self-Assembled Monolayer of 5-Methoxy-2-(Octadecylthio)Benzimidazole in Corrosion Protection of Copper. Electrochim. Acta.

[30] Pareek S., Jain D., Hussain S., Biswas A., Shrivastava R., Parida S.K., Kisan H.K., Lgaz H., Chung I.-M., Behera D. (2019). A New Insight into Corrosion Inhibition Mechanism of Copper in Aerated 3.5 Wt.% NaCl Solution by Eco-Friendly Imidazopyrimidine Dye: Experimental and Theoretical Approach. Chem. Eng. J..

[31] Khaled K.F. (2003). The Inhibition of Benzimidazole Derivatives on Corrosion of Iron in 1 M HCl Solutions. Electrochim. Acta.

[32] Aljourani J., Raeissi K., Golozar M.A. (2009). Benzimidazole and Its Derivatives as Corrosion Inhibitors for Mild Steel in 1M HCl Solution. Corros. Sci..

[33] Chaouiki A., Chafiq M., Rbaa M., Salghi R., Lakhrissi B., Ali I.H., Bashir S., Chung I.-M. (2020). Comprehensive Assessment of Corrosion Inhibition Mechanisms of Novel Benzimidazole Compounds for Mild Steel in HCl: An Experimental and Theoretical Investigation. J. Mol. Liq..

[34] Ech-chihbi E., Adardour M., Ettahiri W., Salim R., Ouakki M., Galai M., Baouid A., Taleb M. (2023). Surface Interactions and Improved Corrosion Resistance of Mild Steel by Addition of New Triazolyl-Benzimidazolone Derivatives in Acidic Environment. J. Mol. Liq..

[35] Rossi A., Hunger A., Kebrle J., Hoffmann K. (1960). Benzimidazol-Derivate und verwandte Heterocyclen V. Die Kondensation von o-Phenylendiamin mit aliphatischen und alicyclischen ?-Ketoestern. Helv. Chim. Acta.

[36] Ibrahim S., Haouas A., Al-Ghulikah H., Mtiraoui H., Jeanneau E., Msaddek M., Hajji M. (2022). Synthesis and Structure of Polycyclic 1,2,3-Triazolylmethyl-Benzimidazole Derivatives—Experimental and Computational Quantum Chemical Studies. J. Mol. Struct..

[37] Adardour M., Zaballos-García E., Loughzail M., Dahaoui S., Baouid A. (2018). Synthesis, Characterization and X-Ray Structure of Heterocyclic Systems Prepared via 1,3-Dipolar Cycloaddition of Nitrile Oxides with Benzimidazolone. J. Mol. Struct..

[38] Antonijević M.M., Milić S.M., Petrović M.B. (2009). Films Formed on Copper Surface in Chloride Media in the Presence of Azoles. Corros. Sci..

[39] Huang H., Wang Z., Gong Y., Gao F., Luo Z., Zhang S., Li H. (2017). Water Soluble Corrosion Inhibitors for Copper in 3.5 Wt% Sodium Chloride Solution. Corros. Sci..

[40] Hsissou R., Benhiba F., Echihi S., Benzidia B., Cherrouf S., Haldhar R., Ahmad Alvi P., Kaya S., Serdaroğlu G., Zarrouk A. (2021). Performance of Curing Epoxy Resin as Potential Anticorrosive Coating for Carbon Steel in 3.5% NaCl Medium: Combining Experimental and Computational Approaches. Chem. Phys. Lett..

[41] Hsissou R., Benhiba F., El Aboubi M., Abbout S., Benzekri Z., Safi Z., Rafik M., Bahaj H., Kaba M., Galai M. (2022). Synthesis and Performance of Two Ecofriendly Epoxy Resins as a Highly Efficient Corrosion Inhibition for Carbon Steel in 1 M HCl Solution: DFT, RDF, FFV and MD Approaches. Chem. Phys. Lett..

[42] Kear G., Barker B.D., Walsh F.C. (2004). Electrochemical Corrosion of Unalloyed Copper in Chloride Media––a Critical Review. Corros. Sci..

[43] Ismail K.M. (2007). Evaluation of Cysteine as Environmentally Friendly Corrosion Inhibitor for Copper in Neutral and Acidic Chloride Solutions. Electrochim. Acta.

[44] Ansari K.R., Quraishi M.A., Singh A. (2014). Schiff’s Base of Pyridyl Substituted Triazoles as New and Effective Corrosion Inhibitors for Mild Steel in Hydrochloric Acid Solution. Corros. Sci..

[45] Chauhan D.S., Ansari K.R., Sorour A.A., Quraishi M.A., Lgaz H., Salghi R. (2018). Thiosemicarbazide and Thiocarbohydrazide Functionalized Chitosan as Ecofriendly Corrosion Inhibitors for Carbon Steel in Hydrochloric Acid Solution. Int. J. Biol. Macromol..

[46] Haque J., Srivastava V., Chauhan D.S., Lgaz H., Quraishi M.A. (2018). Microwave-Induced Synthesis of Chitosan Schiff Bases and Their Application as Novel and Green Corrosion Inhibitors: Experimental and Theoretical Approach. ACS Omega.

[47] Popova A., Raicheva S., Sokolova E., Christov M. (1996). Frequency Dispersion of the Interfacial Impedance at Mild Steel Corrosion in Acid Media in the Presence of Benzimidazole Derivatives. Langmuir.

[48] Popova A., Christov M., Raicheva S., Sokolova E. (2004). Adsorption and Inhibitive Properties of Benzimidazole Derivatives in Acid Mild Steel Corrosion. Corros. Sci..

[49] Jing C., Wang Z., Gong Y., Huang H., Ma Y., Xie H., Li H., Zhang S., Gao F. (2018). Photo and Thermally Stable Branched Corrosion Inhibitors Containing Two Benzotriazole Groups for Copper in 3.5 Wt% Sodium Chloride Solution. Corros. Sci..

[50] Solmaz R., Kardaş G., Çulha M., Yazıcı B., Erbil M. (2008). Investigation of Adsorption and Inhibitive Effect of 2-Mercaptothiazoline on Corrosion of Mild Steel in Hydrochloric Acid Media. Electrochim. Acta.

[51] Idouhli R., N’Ait Ousidi A., Khadiri M.E., Abouelfida A., Ait Itto M.Y., Auhmani A., Benyaich A. (2021). Electrochemical and Theoretical Studies of Some Monoterpenic Thiazolidinones as Corrosion Inhibitors for Steel in Acidic Media. J. Adhes. Sci. Technol..

[52] Yadav D., Mishra S., Shanker V., Haranath D. (2013). Design and Development of Low-Power Driven Hybrid Electroluminescent Lamp from Carbon Nanotube Embedded Phosphor Material. J. Alloys Compd..

[53] Idouhli R., Oukhrib A., Koumya Y., Abouelfida A., Benyaich A., Benharref A. (2018). Inhibitory Effect of Atlas Cedar Essential Oil on the Corrosion of Steel in 1 m HCl. Corros. Rev..

[54] Yoo S.-H., Kim Y.-W., Chung K., Kim N.-K., Kim J.-S. (2013). Corrosion Inhibition Properties of Triazine Derivatives Containing Carboxylic Acid and Amine Groups in 1.0 M HCl Solution. Ind. Eng. Chem. Res..

[55] Corrales Luna M., Le Manh T., Cabrera Sierra R., Medina Flores J.V., Lartundo Rojas L., Arce Estrada E.M. (2019). Study of Corrosion Behavior of API 5L X52 Steel in Sulfuric Acid in the Presence of Ionic Liquid 1-Ethyl 3-Methylimidazolium Thiocyanate as Corrosion Inhibitor. J. Mol. Liq..

[56] Kannan P., Jithinraj P., Natesan M. (2018). Multiphasic Inhibition of Mild Steel Corrosion in H 2 S Gas Environment. Arab. J. Chem..

[57] Guo Y., Chen Z., Zuo Y., Chen Y., Yang W., Xu B. (2018). Ionic Liquids with Two Typical Hydrophobic Anions as Acidic Corrosion Inhibitors. J. Mol. Liq..

[58] James A.O., Iroha N.B. (2022). New Green Inhibitor of Olax Subscorpioidea Root for J55 Carbon Steel Corrosion in 15% HCl: Theoretical, Electrochemical, and Surface Morphological Investigation. Emergent Mater..

[59] Zhang J., Zhang L., Tao G. (2018). A Novel and High-Efficiency Inhibitor of 5-(4-Methoxyphenyl)-3h-1,2-Dithiole-3-Thione for Copper Corrosion Inhibition in Sulfuric Acid at Different Temperatures. J. Mol. Liq..

[60] da Rocha J.C., da Cunha Ponciano Gomes J.A., D’Elia E. (2010). Corrosion Inhibition of Carbon Steel in Hydrochloric Acid Solution by Fruit Peel Aqueous Extracts. Corros. Sci..

[61] Khadiri M., Idouhli R., Bennouna M.A., Aityoub A., Abouelfida A., Benyaïch A. (2021). Contribution to Understanding Synergistic Effect of *Punica Granatum* Extract and Potassium Iodide as Corrosion Inhibitor of S355 Steel. Corros. Rev..

[62] Idouhli R., Koumya Y., Khadiri M., Aityoub A., Abouelfida A., Benyaich A. (2019). Inhibitory effect of Senecio anteuphorbium as green corrosion inhibitor for S300 steel. Int. J. Ind. Chem..

[63] Stanton J.F. (2001). A Chemist’s Guide to Density Functional Theory by Wolfram Koch (German Chemical Society, Frankfurt Am Main) and Max C. Holthausen (Humbolt University Berlin). Wiley-VCH: Weinheim. 2000. Xiv + 294 Pp. $79.95. ISBN 3-527-29918-1. J. Am. Chem. Soc..

[64] (2021). SAINT a Program for Integration of Raw X-ray Diffraction Intensities.

[65] Krause L., Herbst-Irmer R., Sheldrick G.M., Stalke D. (2015). SADABS Program for Absorption Correction and Merging of Equivalent Reflections. J. Appl. Cryst..

[66] Sheldrick G.M. (2015). SHELXT, a Dual Space Structure Solution, Program. Acta Crystallographica..

[67] Sheldrick G.M. (2015). SHELXL, a Full-Matrix, Least-Squares Structure Refinement Program. Acta Crystallographica..

[68] Aslam R. (2023). Potentiodynamic Polarization Methods for Corrosion Measurement. Electrochemical and Analytical Techniques for Sustainable Corrosion Monitoring.

[69] Zhang M., Liu Y., Li D., Cui X., Wang L., Li L., Wang K. (2023). Electrochemical Impedance Spectroscopy: A New Chapter in the Fast and Accurate Estimation of the State of Health for Lithium-Ion Batteries. Energies.

[70] Xiong R., Tian J., Shen W., Lu J., Sun F. (2023). Semi-Supervised Estimation of Capacity Degradation for Lithium Ion Batteries with Electrochemical Impedance Spectroscopy. J. Energy Chem..

[71] Frisch M.J., Trucks G.W., Schlegel H.B., Scuseria G.E., Robb M.A., Cheeseman J.R., Scalmani G., Barone V., Mennucci B., Petersson G.A. (2009). Hada GAUSSIAN 09 R’evision.

[72] Becke A.D. (1988). Density-Functional Exchange-Energy Approximation with Correct Asymptotic-Behavior. Phys. Rev. A.

[73] Becke A.D. (1993). Density-functional Thermochemistry. III. The Role of Exact Exchange. J. Chem. Phys..

[74] Lee C., Yang W., Parr R.G. (1988). Development of the Colle-Salvetti Correlation-Energy Formula into a Functional of the Electron Density. Phys. Rev. B.

[75] Zhang Q.H., Hou B.S., Zhang G.A. (2020). Inhibitive and Adsorption Behavior of Thiadiazole Derivatives on Carbon Steel Corrosion in CO2-Saturated Oilfield Produced Water: Effect of Substituent Group on Efficiency. J. Colloid Interface Sci..

[76] Tan B., Zhang S., Qiang Y., Li W., Li H., Feng L., Guo L., Xu C., Chen S., Zhang G. (2020). Experimental and Theoretical Studies on the Inhibition Properties of Three Diphenyl Disulfide Derivatives on Copper Corrosion in Acid Medium. J. Mol. Liq..

[77] Oubaaqa M., Ouakki M., Rbaa M., Benhiba F., Galai M., Idouhli R., Maatallah M., Jarid A., Warad I., Lakhrissi B. (2022). Experimental and Theoretical Investigation of Corrosion Inhibition Effect of Two 8-Hydroxyquinoline Carbonitrile Derivatives on Mild Steel in 1 M HCl Solution. J. Phys. Chem. Solids.

[78] Vorberg S., Tetko I.V. (2014). Modeling the Biodegradability of Chemical Compounds Using the Online CHEmical Modeling Environment (OCHEM). Mol. Inf..

[79] Li X., Zhang Y., Li H., Zhao Y. (2017). Modeling of the hERG K+ Channel Blockage Using Online Chemical Database and Modeling Environment (OCHEM). Mol. Inf..

