# Supplementary material for: Exploring the Efficacy of Benzimidazolone Derivative as Corrosion Inhibitors for Copper in a 3.5 wt.% NaCl Solution: A Comprehensive Experimental and Theoretical Investigation
Source: Molecules. 2023 Oct 6;28(19):6948. doi: 10.3390/molecules28196948 (PMC10574370; doi:10.3390/molecules28196948)

Supplementary Data

Exploring the Efficacy of Benzimidazolone Derivative as Corrosion Inhibitors for Copper in a 3.5 wt.% NaCl Solution: A Comprehensive Experimental and Theoretical Investigation

Mohamed Adardour [1,*], Mohammed Lasri [2], Marouane Ait Lahcen [1], Mohamed Maatallah [1], Rachid Idouhli [2], Mohamed M. Alanazi [3], Sanae Lahmidi [4], Abdesselam Abouelfida [2], Joel T. Mague [5] and Abdesselam Baouid [1]

[1]Laboratory of Chemistry Molecular, Department of Chemistry, Faculty of Sciences Semlalia, Cadi Ayyad University, B.P. 2390, Marrakech 40001, Morocco; m.aitlahcen.ced@uca.ac.ma (M.A.L.); m.maatallah@uca.ma (M.M.); baouid@uca.ac.ma (A.B.)
[2]Applied Chemistry and Biomass Laboratory, Department of Chemistry, Faculty of Sciences Semlalia, Cadi Ayyad University, B.P. 2390, Marrakech 40001, Morocco; m.lasri.ced@uca.ac.ma (M.L.); rachid.idouhli@uca.ac.ma (R.I.); abouelfida@uca.ac.ma (A.A.)
[3]Department of Pharmaceutical Chemistry, College of Pharmacy, King Saud University, P.O. Box 2457, Riyadh 11451, Saudi Arabia; mmalanazi@ksu.edu.sa
[4]Laboratory of Heterocyclic Organic Chemistry, Department of Chemistry, Faculty of Sciences, Mohammed V University in Rabat, Rabat 10106, Morocco; lahmidi_sanae@yahoo.fr
[5]Department of Chemistry, Tulane University, New Orleans, LA 70118, USA; joelt@tulane.edu
* Correspondence: mohamed.adardour@ced.uca.ac.ma

Figurer S1. ^{1}H NMR of Compound CHBI

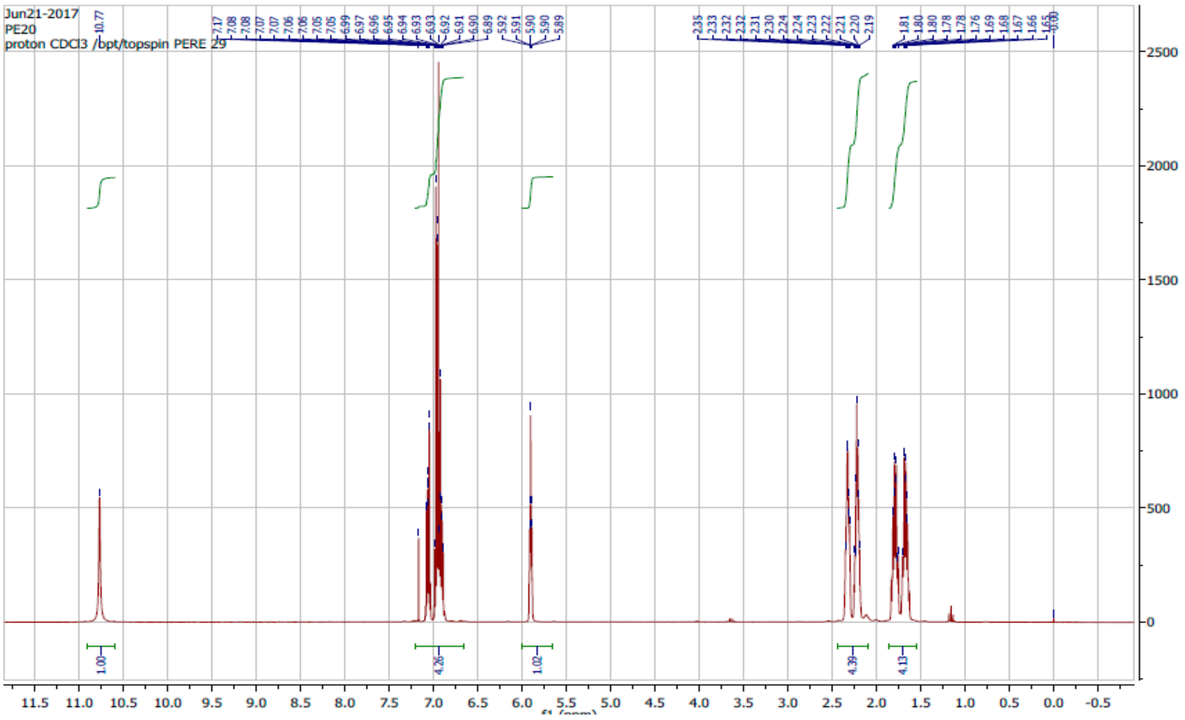

Figure S2. ^{13}C NMR of Compound CHBI

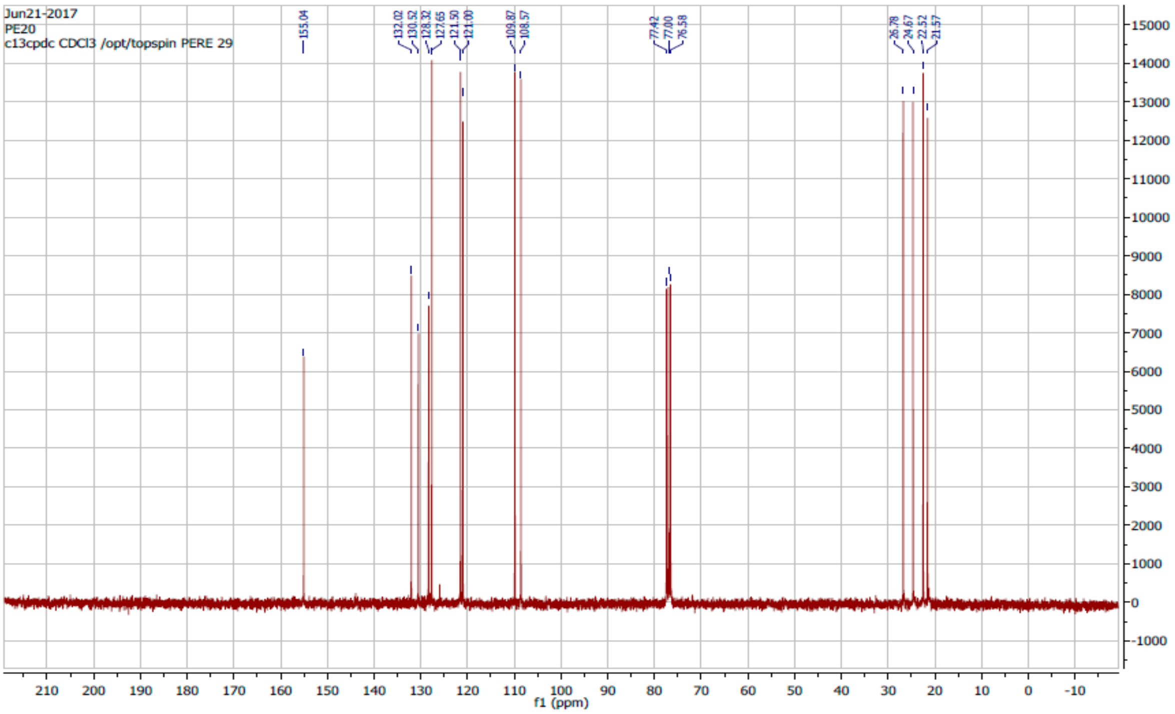

Figure S3. HRMS of Compound CHBI

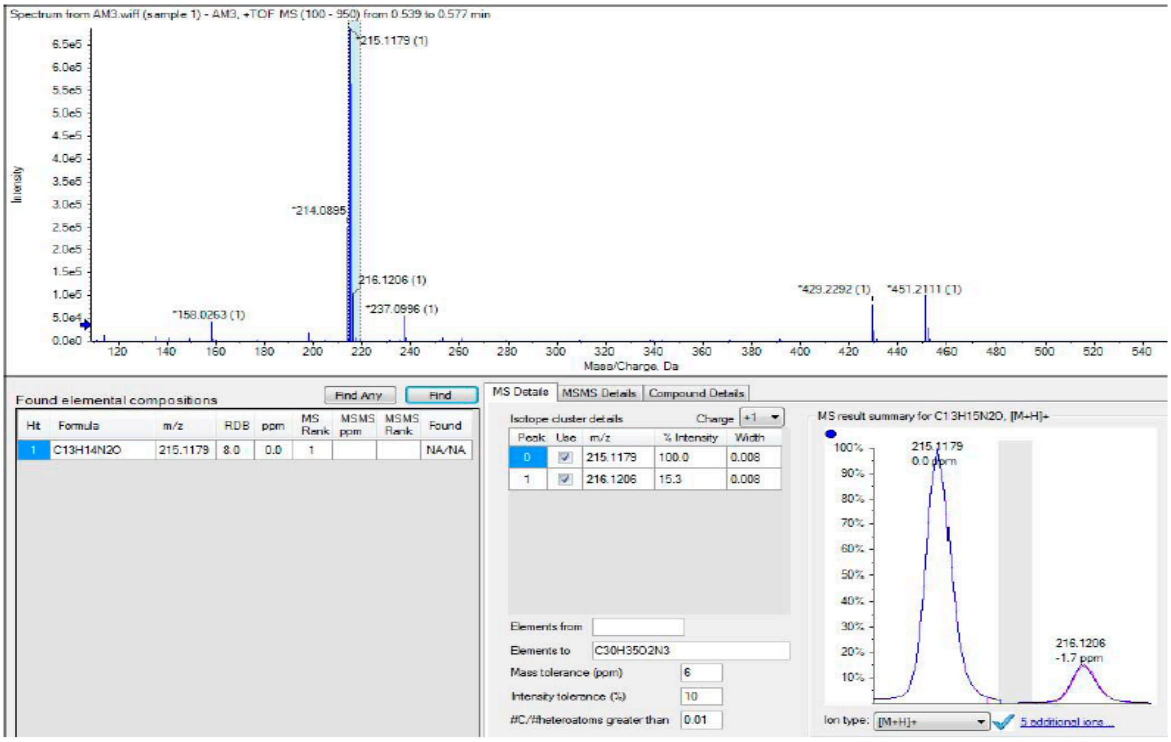

Supplement: Supplementary file 1 [file molecules-28-06948-s001.zip › molecules-2628831-supplementary.pdf]
